# Contemporary Management of Cardiogenic Shock Complicating Acute Myocardial Infarction

**DOI:** 10.3390/jcm12062184

**Published:** 2023-03-11

**Authors:** Leonardo De Luca, Raffaella Mistrulli, Riccardo Scirpa, Holger Thiele, Giuseppe De Luca

**Affiliations:** 1 Department of Cardio-Thoracic and Vascular Medicine and Surgery, Division of Cardiology, A.O. San Camillo-Forlanini, 00152 Rome, Italy; 2 Faculty of Medicine and Dentistry, UniCamillus-Saint Camillus International University of Health Sciences, 00131 Rome, Italy; 3 Department of Cardiology, Heart Center Leipzig, University of Leipzig, 04289 Leipzig, Germany; 4 Division of Cardiology, AOU “Policlinico G. Martino”, Department of Clinical and Experimental Medicine, University of Messina, 98166 Messina, Italy; 5 Division of Cardiology, IRCCS Hospital Galeazzi-Sant’Ambrogio, 20161 Milan, Italy

**Keywords:** acute myocardial infarction, cardiogenic shock, mechanical circulatory supports

## Abstract

Despite an improvement in pharmacological therapies and mechanical reperfusion, the outcome of patients with acute myocardial infarction (AMI) is still suboptimal, especially in patients with cardiogenic shock (CS). The incidence of CS accounts for 3–15% of AMI cases, with mortality rates of 40% to 50%. In contrast to a large number of trials conducted in patients with AMI without CS, there is limited evidence-based scientific knowledge in the CS setting. Therefore, recommendations and actual treatments are often based on registry data. Similarly, knowledge of the available options in terms of temporary mechanical circulatory support (MCS) devices is not equally widespread, leading to an underutilisation or even overutilisation in different regions/countries of these treatment options and nonuniformity in the management of CS. The aim of this article is to provide a critical overview of the available literature on the management of CS as a complication of AMI, summarising the most recent evidence on revascularisation strategies, pharmacological treatments and MCS use.

## 1. Introduction

Cardiogenic shock (CS) is characterised by a state of tissue hypoperfusion secondary to reduced cardiac output (CO) in the presence of adequate intravascular volume [1]. Acute myocardial infarction (AMI) with left ventricular (LV) dysfunction remains the most frequent cause of CS. However, other causes of CS are rising. Currently, the incidence of CS accounts for 3–15% of AMI cases. Despite the introduction of myocardial revascularisation therapy and an overall improvement in mechanical reperfusion strategies [2,3,4], CS remains the leading cause of in-hospital death in patients with AMI, with an in-hospital and 1-year mortality rate of 40% and 50%, respectively [5]. Some recent registries reported an increased mortality rate, which may be related to changes in the mean age and risk profiles of CS patients [6,7].

In contrast to a large number of trials conducted in patients with AMI without CS, there is limited evidence-based scientific knowledge in the CS setting. Therefore, recommendations and actual treatments are often based on registry data. Similarly, knowledge of the available options in terms of temporary mechanical circulatory support (MCS) devices is not equally widespread, leading to an underutilisation or even overutilisation in different regions/countries of these treatment options and nonuniformity in the management of CS [8]. This review focuses on the management of CS as a complication of AMI, summarising the most recent evidence on revascularisation strategies, pharmacological treatments and MCS use.

## 2. Definition and Pathophysiology

CS results from impaired heart pumping function leading to inadequate delivery of blood to the tissue to meet metabolic demands [8]. The diagnosis of CS can be made on the basis of clinical criteria such as persistent hypotension (systolic blood pressure <90 mmHg) in the presence of adequate intravascular volume, accompanied by clinical signs such as cold extremities, oliguria, mental confusion, dizziness and narrow pulse pressure, or laboratory signs of hypoperfusion (metabolic acidosis, elevated lactate and newly elevated creatinine). Although not mandatory in clinical practice, objective haemodynamic parameters, such as reduced cardiac index ≤2.2 L/min/m^2^ and elevated pulmonary capillary wedge pressure (PCWP) ≥15 mmHg, are useful for a correct diagnosis. The definitions applied in selected major randomised clinical trials (RCTs) and guidelines are shown in Table 1

In general, severe myocardial ischaemia leads to an increase in LV filling pressure and then to a reduction in CO with systemic hypotension and hypoperfusion of peripheral tissues. Cardiac function is impaired due to a reduction in coronary perfusion and myocardial ischaemia, leading to worsening of systolic and diastolic dysfunction in a vicious cycle format. Increased LV filling pressures raise the hydrostatic pressure of the pulmonary capillaries and lead to congestion and pulmonary oedema, exacerbating hypoxaemia [1,8]. Renal hypoperfusion triggers the activation of the renin–angiotensin–aldosterone system, promoting sodium and water retention. Vasoconstriction of the splanchnic bed, caused by the sympathetic activation, mobilises blood to the central circulation but may trigger a barrier disruption in the mesentery microcirculation, with translocation of bacteria or bacterial toxins and possible subsequent septic reaction. In addition, a severe acute inflammatory response syndrome, characterised by the activation of various inflammatory cascades (generation of free radicals, complement formation, cytokine release) and cellular components (leukocytes, platelets, monocytes and endothelial cells), has been described in a substantial percentage of CS patients. Hence, the initial compensatory systemic vasoconstriction may subsequently be contrasted by pathological vasodilation due to inflammatory reactions. Impaired mental status, as a consequence of cerebral hypoperfusion, is common at the onset of CS, and it is associated with a worse prognosis [1].

**Figure 1:**
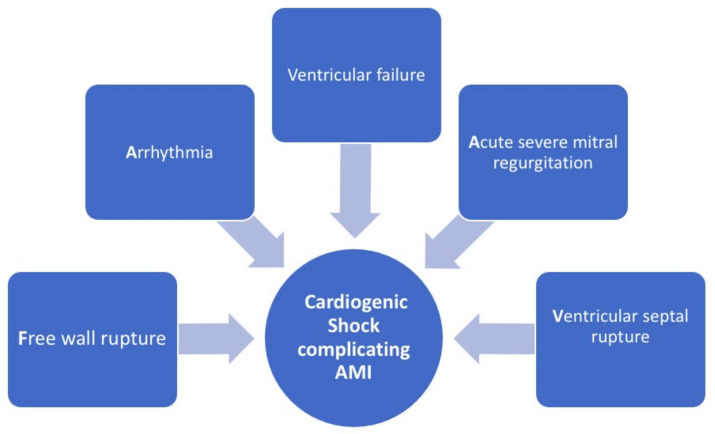
Causes of CS complicating AMI Ventricular failure subsequent to AMI remains the most frequent cause of CS. Mechanical complications of AMI represent less frequent causes (acute severe mitral regurgitation, ventricular septal rupture, free wall rupture): AMI, acute myocardial infarction; CS, cardiogenic shock.

The large ischaemic territory often encountered in these patients [9,10], the severe acute inflammatory and prothrombotic response and the increase in vasoactive mediators may all explain the higher occurrence of impaired reperfusion and no-reflow linearly observed with the degree of haemodynamic impairment [11,12], which may, in turn, further worsen cardiac haemodynamic and prognosis. There are also other pathophysiologic reasons, such as acute mitral regurgitation, ventricular septal defect or predominant right ventricular (RV) infarction with CS. RV infarction and failure are associated with increased RV and right atrial pressure. The classical triad is: hypotension, elevated jugular venous pressure and tachycardia in the presence of normal oxygen saturation. Due to ventricular interdependence, RV failure may also impair LV filling, resulting in reduced CO [13] (Figure 1).

## 3. Clinical Assessment and Diagnosis

The clinical presentation is marked by signs of hypoperfusion, such as cool or mottled extremities, altered mental status and low urine output, generally associated with hypotension and tachycardia, as well as signs of elevated filling pressure, such as turgor of the jugular veins, orthopnoea and pulmonary rales. Moreover, low CO can cause organ damage, leading to kidney or liver failure up to multiorgan failure and death [13]. Elevation in serum lactate is a sensitive laboratory marker of hypoperfusion, and serial assessments are of utmost importance to evaluate the response to the therapy [14]. Since AMI is the leading cause of CS, an electrocardiogram should be carried out as soon as possible. Besides laboratory testing and chest X-ray, it is essential to perform an echocardiogram in order to exclude mechanical complications and to rapidly evaluate biventricular and valvular function. Although not essential to the diagnosis, right heart catheterisation (RHC) may be important in select cases to confirm the diagnosis, identify the causes of shock, provide prognostic information and guide vasoactive therapy [15]. Some recent retrospective observational studies suggest a reduction in the mortality rate correlated with the use of RHC in patients with AMI-related CS treated with MCS [16,17]. Even so, there is a lack of evidence and the only randomised trial addressing this issue, in which patients with CS were excluded, showed that RHC did not affect overall mortality and hospitalisation in decompensated heart failure [18].

## 4. Classification and Prognosis

**Figure 2:**
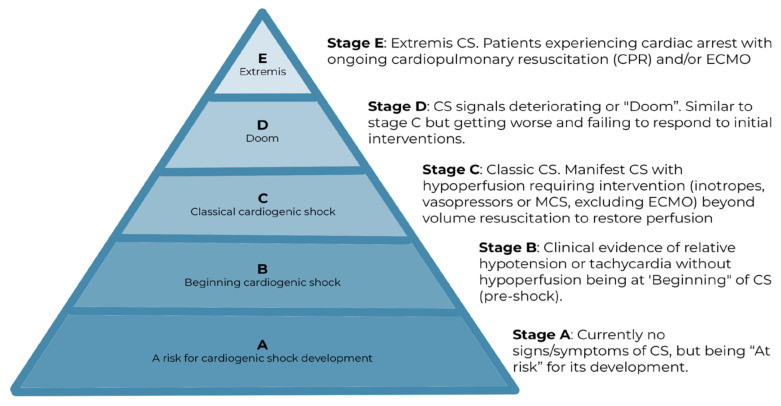
The pyramid of CS classification, according to the Society of Cardiovascular Angiography and Interventions (SCAI).

Recently, the Society of Cardiovascular Angiography and Interventions (SCAI) proposed a simple and effective classification for CS [19]. It consists of five stages with increasing risk of mortality. Stage A includes patients at risk of developing CS, while stage B, or preshock, encompasses those with relative tachycardia or hypotension without signs of hypoperfusion; these patients must be strictly monitored and treated in order to prevent the development of a classic state of shock (stage C). Stage D includes patients who do not respond to initial stabilisation measures, become worse and require advanced hemodynamic support. Finally, stage E is defined by circulatory collapse, often with refractory cardiac arrest (CA), or the need to be supported by multiple acute interventions, including extracorporeal membrane oxygenation (ECMO) (Figure 2). The SCAI classification has been shown to provide robust mortality risk stratification, allowing optimal selection of patients requiring advanced support and, at the same time, possibly avoiding futile interventions [20]. However, currently, the risk prediction in the different stages provides huge heterogeneity and a large range of mortality risk, which precludes individual risk assessment. Whether the refined SCAI shock classification is able to address this needs further validation studies [21].

Several scores have been developed for prognosis assessment in patients with CS complicating AMI, including the IABP-SHOCK II score [22], derived from the IABP-SHOCK II trial [23], which has been both internally and externally validated and is based on six simple clinical variables: age, history of stroke, glucose, creatinine, arterial lactate and TIMI flow grade <3 after percutaneous coronary intervention (PCI). Recently, Ceglarek et al. [24] proposed the more objective CLIP score, a biomarker risk score derived from the CULPRIT-SHOCK trial [25] and externally validated using the IABP-SHOCK II trial [18]; this score, which includes four routinely available blood biomarkers (cystatin C, lactate, interleukin-6, NT-proBNP), outperformed the IABP-SHOCK II score in prognostication, yielding a higher C statistic.

Among patients with ST-segment elevation AMI (STEMI) treated with primary PCI, identifying those with a high risk of in-hospital development of CS at this stage may have the greatest effect on prognosis. In this setting, the ORBI score [26], comprising 11 patient- or procedure-related variables, has been proposed as an effective tool to recognise high-risk patients that may benefit from early, more aggressive therapies [27].

Finally, as mentioned above, risk stratification is fundamental for clinical decision-making, even in refractory CS, in which case we see very high short-term mortality despite extensive haemodynamic support, generally provided by ECMO [28]. In a view to preventing futility, Schmidt et al. [29] proposed a score to predict in-hospital survival for patients receiving ECMO for refractory CS, showing a better performance than other severity scores, such as the APACHE III and the SOFA score.

Despite significant improvements in in-hospital prognosis from the 1980s to the early 2000s, in recent years, there has been a plateau in the short-term mortality rate [30]. Moreover, the development of CS in the ‘prehospital’ phase (before hospital arrival for AMI) is associated with a worse prognosis in comparison to patients who deteriorate during hospitalisation [31]. In the SHOCK trial, the leading causes of death in CS were cardiac, especially in the first 30 days, mostly due to pump failure. After the first 30 days, almost half of deaths had a noncardiac cause and were associated with signs of inflammation with a lower systemic vascular resistance index [25].

Regarding long-term prognosis, around half of CS-complicating-AMI survivors have a hospital readmission, mostly due to heart failure or myocardial ischaemia. They have higher rates of death and rehospitalisation than other AMI patients, especially in the first 60 days; however, after this early vulnerable postdischarge period, the outcomes are similar between patients with and without CS [32].

## 5. Management

### 5.1. Coronary Artery Revascularisation

A strategy of early revascularisation is the only measure that has proved to significantly reduce overall mortality in patients with CS due to AMI [33]. Indeed, the SHOCK trial [34] showed that early revascularisation, either by PCI or coronary artery bypass graft (CABG), resulted in lower 6-month mortality from all causes compared with initial intensive medical therapy, followed by no or late in-hospital revascularisation. These results were confirmed in a longer follow-up, with a 67% relative improvement in 6-year survival [35,36]. Since then, the widespread use of early revascularisation has led to a decrease in mortality from 70–80%, as reported in historical cohorts [37], to 40–50% in contemporary registries [38,39].

Currently, emergency PCI is indicated in patients with CS complicating AMI with a class I recommendation, irrespective of the time delay [40]. A recent study [41] highlighted that few patients with CS reached the guideline-recommended time target and that a delay in revascularisation is associated with a lower survival rate. Due to its limited efficacy, fibrinolytic therapy should be considered in STEMI only when timely PCI is unavailable [42].

**Figure 3:**
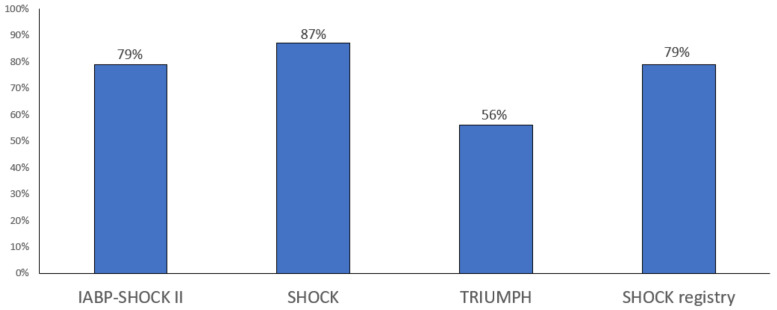
Prevalence multivessel disease in infarct-related shock.

Most CS-complicating-AMI patients have multivessel disease and have higher mortality compared with patients with single-vessel disease [43]. The prevalence of multivessel disease in these patients ranges from 56% in the TRIUMPH trial (Tilarginine Acetate Injection in a Randomized International Study in Unstable MI Patients with Cardiogenic Shock) to 87% in the SHOCK trial [34,44] (Figure 3). However, the number of diseased vessels was found to be associated with worse 1-year survival only in patients who underwent initial medical stabilisation after adjustment for key clinical factors [45].

Although rarely performed, emergency CABG should be a valid alternative to PCI in these patients, having shown similar mortality rates, and may be preferred when complete PCI is not possible [45].

**Figure 4:**
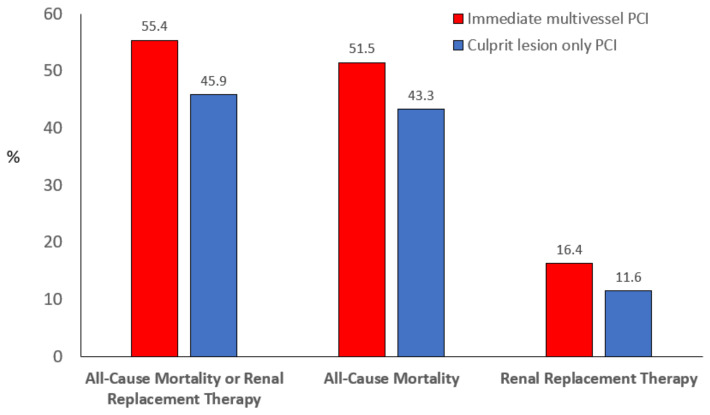
Main results of CULPRIT-SHOCK trial [17].

The CULPRIT-SHOCK (Percutaneous Coronary Intervention Strategies with Acute Myocardial Infarction and Cardiogenic Shock) trial [25] is the largest randomised trial conducted in CS-complicating-AMI patients; it compared two revascularisation strategies: PCI of the culprit lesion only, with the option of a staged revascularisation of nonculprit lesions, or immediate multivessel PCI. At 30 days, the risk of a composite of death or renal replacement therapy was lower in the first group, largely due to a significant reduction in mortality among patients who underwent culprit-lesion-only PCI (Figure 4). Although staged revascularisation was encouraged, it was performed only in 17.7% of patients in the culprit-lesion-only PCI group. The difference in the composite endpoint was maintained at 1-year follow-up, whereas the difference in mortality was attenuated between the two groups [46]. Moreover, patients achieving complete revascularisation showed the most favourable prognosis. Considering all these findings, in the acute phase, PCI should be limited to the culprit lesion with possible staged revascularisation at a later time [47].

### 5.2. Pharmacologic Therapies

Fluids, diuretics, vasopressor drugs and inotropes are essential for hemodynamic stabilisation and improvement in CO in CS, but evidence supporting their prognostic impact is modest [48].

The first-line pharmacological treatment is represented by fluid therapy, which, to date, represents the only therapy with a class Ia recommendation on current guidelines [49]. The “fluid challenge” technique, characterised by the use of >200 mL of fluids (usually colloids or ringer lactate) infused for 15–30 min in the absence of signs of volume overload, has the ability to improve systemic blood pressure in the event of patient responsiveness, at limited risk of side effects. With a class IIa recommendation, guidelines recommend the use of vasopressors to increase blood pressure and perfusion of vital organs, and in class IIb, inotropes to increase cardiac index and blood pressure and to improve peripheral perfusion [50]. Although catecholamines are used in more than 90% of patients with CS, evidence from RCTs is limited and controversial [3,51]. The choice of vasopressor/inotrope is generally empirical but should also be guided by the possible hemodynamic effects of each drug. Dobutamine is a potent agonist of the ß1-receptors (Rs) and, with lower affinity, also to the ß2- Rs and α1-Rs [52]. Moreover, in addition to the predominant positive inotropic effect, it induces peripheral vasodilation. Indeed, for any given increase in cardiac contractility, an increase in heart rate and blood pressure is lower with dobutamine than with dopamine or norepinephrine (NA), further reflecting dobutamine’s selectivity for ß1 over other Rs. Conversely, it increases myocardial O_2_ consumption, induces arrhythmias and prolongs high infusions that may induce tachyphylaxis and eosinophilic hypersensitivity. NA has a predominant effect on α1-Rs and is the recommended first-line vasopressor agent [48,49,50,51,52]. In an RCT comparing the vasoconstrictors dopamine and NA in CS, more arrhythmic and fatal events occurred among patients treated with dopamine at 28 days [53]. In another study comparing NA and epinephrine in 57 patients with CS due to AMI, NA was associated with a lower incidence of refractory CS than epinephrine. Notably, epinephrine also resulted in a significant increase in heart rate and some unfavourable metabolic changes, such as increased double product and lactic acidosis [53]. Although epinephrine and NA have a similar effect on blood pressure, epinephrine (but not NA) increases heart rate and myocardial oxygen consumption due to its strong activation of ß2-Rs and ß1-Rs [52].

In a retrospective population-based study of the Cardshock trial [54], epinephrine was independently associated with increased 90-day mortality (the primary outcome) and worsening of cardiac and renal biomarkers during the first few days. The result was independent of prior CA (39% of patients treated with epinephrine). Dobutamine and levosimendan were the most commonly used inotropes (49% and 24%), and there was no difference in mortality whether NA was combined with dobutamine or levosimendan [54]. Levosimendan is an inotropic agent that sensitises troponin C to calcium, improving myocardial contractility, and promotes the opening of ATP-dependent potassium channels in the vascular smooth muscle, resulting in coronary and secondary systemic vasodilation [52,55]. Although levosimendan is an inotrope studied in the context of patients with acute heart failure, clinical evidence in the context of CS is limited. In a study of 10 patients with CS post-AMI or cardiac surgery, levosimendan was infused at a standard dose (0.1 microg/kg/min) for 24 h without initial bolus and with NA infusion to maintain a mean arterial pressure of ≥65 mmHg. During the infusion, there was an increase in cardiac index and a reduction in peripheral resistance [56].

Phosphodiesterase type 3 inhibitors result in increased intracellular levels of cyclic AMP and act both as an inotrope and a peripheral and pulmonary vasodilator. Phosphodiesterase type 3 inhibitors are useful, especially in patients on chronic beta-blocker treatment, as they act independently of beta-receptors [52], but in those patients with CS secondary to AMI, they are not recommended. Patients enrolled in the OPTIME-CHF (Outcomes of a Prospective Trial of Intravenous Milrinone for Exacerbations of Chronic Heart Failure) trial with CS secondary to AMI and treated with intravenous milrinone tended to have worse outcomes than those treated with placebo in terms of the primary endpoint (days hospitalised from cardiovascular causes within 60 days) and the composite of death or rehospitalisation (42% vs. 36% for placebo, *p* = 0.01 for interaction) [57,58]. Moreover, milrinone led to more hypotension and more arrhythmias.

In some clinical settings such as severe acute mitral regurgitation the use of vasodilators such as sodium nitroprusside and nitroglycerine may be useful. The expected effect is a reduction in peripheral and pulmonary vascular resistance with a reduction in ventricular filling pressures and an improvement in CO [59].

Two recent randomised trials [60,61] tested the hypothesis of whether mild or moderate hypothermia may provide benefits in the outcome in patients with CS treated with a mechanical support, reaching negative results.

The largest trial [61], the HYPO-ECMO trial, randomly assigned a total of 374 patients (eligible if they had been endotracheally intubated and were receiving venoarterial ECMO for CS for <6 h) to early moderate hypothermia (33–34 °C; n = 168) for 24 h or strict normothermia (36–37 °C; n = 166). At 30 days, 71 patients (42%) in the moderate hypothermia group had died vs. 84 patients (51%) in the normothermia group (adjusted odds ratio 0.71, 95% CI 0.45 to 1.13, *p* = 0.15).

### 5.3. Mechanical Circulatory Support Devices

The rationale for the use of temporary MCS is to increase CO and tissue perfusion when drug therapies are not sufficient. The final goal is to prevent multiorgan failure and allow the transition to functional recovery of the heart or to long-term MCS. A serial cross-sectional study showed that the use of short-term MCS in the United States increased rapidly (from 2004 to 2011) with contrasting results in terms of mortality [62,63]. Despite the growing number of devices available for short-term MCS in patients with CS (Table 2) and their increasing use, data from RCTs on their safety and efficacy, indications by type and optimal timing of placement are currently limited.

### 5.4. Intra-Aortic Balloon Pump

The IABP is the most widely used device because it is cost-effective and easy to place, with few complications and wide clinical experience; however, there are no documented significant beneficial effects in CO compared with standard medical therapy [64]. A report from the Shock Trial Registry showed that patients with CS treated with IABP had significantly lower in-hospital mortality compared with usual care [65]. On the other hand, from the NRMI-2 (National Registry of Myocardial Infarction 2) registry, the use of IABP was not associated with any benefit in patients with CS undergoing PCI [66]. These results are in line with evidence from the multicentre randomised IABP-SHOCK II trial, the largest trial on the use of IABP in patients with AMI complicated by CS. Indeed, the use of IABP did not significantly reduce 30-day mortality in patients with CS complicating AMI and early PCI planned [23]. No subgroups could be identified with a potential advantage of IABP support, and the 1-year and 6-year follow-up analyses confirmed these negative findings, with a comparable mortality rate between the IABP and the control group [67,68]. Consequently, the results of IABP-SHOCK II influenced recent ESC revascularisation guidelines with the further downgrading of the IABP from a class IC to a class IIIB recommendation for routine use in CS [40].

### 5.5. Percutaneous Ventricular Assist Devices

Impella (Abiomed Inc., Danvers, MA, USA) is a class of device consisting of a catheter-based intravascular microaxial blood pump capable of providing up to 5.5 L of CO and rapidly reducing LV preload [69]. Impella can be maintained in situ for up to 5–7 days (30 days for Impella 5.5), with some differences based on specific models, and its function is not affected by heart rate or residual LV contractile function. There are few data regarding Impella use in CS, so there are limited recommendations in the guidelines [40], raising the possibility of variability in the selection and use of these devices.

The ISAR-SHOCK (Impella LP2.5 vs. IABP in Cardiogenic SHOCK) was the first study to test this technology in a randomised manner and aimed to test the hypothesis that Impella LP2.5 provides superior hemodynamic support compared with IABP in patients with CS caused by AMI. The primary endpoint was the change in the cardiac index from baseline to 30 min after implantation. Although the cardiac index after 30 min of support was significantly increased in patients with Impella LP2.5 compared with those with IABP, the overall 30-day mortality was similar between the two treatment arms [69].

The use of Impella CP was not associated with reduced 30-day mortality compared with IABP also in the IMPRESS-in-Severe-SHOCK (IMPella versus IABP Reduces mortality in STEMI patients treated with primary PCI in Severe cardiogenic SHOCK) trial, an RCT involving mechanically ventilated patients with CS complicating AMI. A trend toward lower 30-day mortality was observed if therapy with Impella CP or IABP was initiated before primary PCI and in patients who did not have traumatic injuries [70]. A meta-analysis aimed to investigate the efficacy and safety of percutaneous active MCS (Impella and Tandem HeartTM) compared with IABP. The analysis included 148 patients with CS due to AMI (four RCTs, two of which are the aforementioned studies). The 30-day mortality rate was similar in patients treated with active MCS in comparison to those undergoing IABP, despite the initial beneficial effects on mean arterial pressure and arterial lactate. In addition, the rate of bleeding was significantly increased in temporary MCS compared with IABP [71]. Schrage et al. [72], in a retrospective analysis including a matched control analysis, confirmed these scarce results on 30-day mortality in addition to a significant increase in the rates of severe or life-threatening bleeding (8.5% versus 3.0%, *p* < 0.01) and peripheral vascular complications (9.8% versus 3.8%, *p* = 0.01) in the Impella group compared with IABP controls.

Similar results have been observed in a retrospective analysis by Amin et al. [73] and in other matched control studies comparing IABP and Impella, with most of them reaching negative conclusions, with even harmful signals with Impella in addition to the higher initial costs [63,74,75].

A large randomised trial is ongoing and evaluating the benefits of preprocedural implantation of Impella LP 2.5 in 330 STEMI patients complicated by CS patients undergoing percutaneous intervention. The results of this trial will hopefully provide more conclusive data on the use of the Impella LP 2.5 device and, more generally, on MCS in this population [76].

The Impella RD is a right ventricular support device consisting of a catheter placed in the femoral vein to the pulmonary valve. In the RECOVER RIGHT trial [77], the Impella RD device was safe, easy to deploy and reliably resulted in an immediate hemodynamic benefit (an increase in the cardiac index and a decrease in central venous pressure), but larger studies are needed to evaluate its efficacy.

It has recently been hypothesised that preprocedural LV unloading before reperfusion by reducing intraventricular pressure may provide significant benefits in terms of coronary reperfusion, infarct size and clinical outcome [78]. The expected benefits should certainly be large and outweigh the deleterious effects of delayed reperfusion due to the insertion of the mechanical supporting device (Impella) and the additional time needed to wait for an effective LV unloading (30 min from insertion) before reperfusion [79]. An ongoing randomised trial will provide more insights into this novel therapeutic approach and its clinical benefits [80].

### 5.6. Extracorporeal Life Support Systems

Venoarterial (VA)-ECMO has been increasingly used in the setting of CS in the last two decades [81].The main reason for this trend is that VA-ECMO, unlike other temporary MCS devices, provides complete cardiopulmonary support, addressing both right and LV dysfunction, systemic oxygenation and acid–base balance [82]. This device consists of a venous cannula that drains the blood from a central vein (usually the femoral vein) and an arterial cannula that pumps the blood in a central artery (usually the femoral artery), as well as a centrifugal pump, an oxygenator and a heat exchanger.

The retrograde flow through the aorta can cause a marked increase in the afterload, leading to a rise in left ventricular end-diastolic pressure and potentially impairing LV function. For this reason, it is often necessary to unload left LV with the use of inotropes or percutaneous MCS, such as IABP or Impella, especially in the case of severe LV systolic dysfunction [83]. In a recent meta-analysis of observational studies, LV unloading during VA-ECMO, mainly through IABP, was shown to be associated with lower mortality at the expense of a higher rate of haemolysis [84], suggesting a potential benefit of this approach in selected patients.

The main indications for VA-ECMO are advanced CS, refractory ventricular tachycardia and refractory CA. Regarding the latter indication, so-called extracorporeal cardiopulmonary resuscitation was found to be related to both short-term and long-term survival benefits in an observational study after a propensity analysis [85]. The benefits in survival with early ECMO implantation in refractory OHCA have been confirmed in a small single-centre randomised trial including 30 patients [86]. However, the largest trial so far conducted by the PRAGUE OHCA Group [87] including 256 patients missed the primary endpoint. Patients were randomly assigned to an invasive strategy (n = 124) (mechanical compression, followed by intra-arrest transport to a cardiac centre for ECPR and immediate invasive assessment and treatment) or standard strategy (regular advanced cardiac life support was continued on-site) (n = 132). The trial was stopped at the recommendation of the data and safety monitoring board when prespecified criteria for futility were met. In the main analysis, 39 patients (31.5%) in the invasive strategy group and 29 (22.0%) in the standard strategy group survived to 180 days with a good neurologic outcome (primary study endpoint, *p* = 0.09). At 30 days, neurologic recovery had occurred in 38 patients (30.6%) in the invasive strategy group and in 24 (18.2%) in the standard strategy group (*p* = 0.02), and cardiac recovery had occurred in 54 (43.5%) and 45 (34.1%) patients, respectively (*p* = 0.12). Bleeding occurred more frequently in the invasive strategy vs. standard strategy group (31% vs. 15%, respectively).

Evidence for VA-ECMO use in CS is mostly limited to observational data [88], and the optimal timing for its use is still unclear. The ECMO-CS randomised study compared the immediate implementation of VA-ECMO vs. an initially conservative therapy (allowing downstream use of VA-ECMO) in patients with rapidly deteriorating CS. The immediate approach did not improve clinical outcomes at 30 days over the conservative strategy [89]. The only small randomised study on CS-complicating-AMI patients, although underpowered, failed to demonstrate an impact of ECMO on LV ejection fraction or all-cause mortality at 30 days [90].

A meta-analysis of nonrandomised studies [91] highlighted increased survival and favourable neurological outcomes in ECMO patients in the setting of refractory CA, while in CS patients, ECMO was associated with higher survival compared with IABP but not compared with Impella or Tandem Heart. On the other hand, another meta-analysis focusing on ACS patients presenting with CS or CA treated with ECMO showed a high rate of short-term mortality, reaching 58% in the first month after the acute event [92]. Furthermore, ECMO is associated with a high rate of complications, such as limb ischaemia, stroke, thrombosis, renal injury, bleeding and infections [93], testifying to the need for a careful selection of patients and an accurate risk–benefit analysis when the initiation of ECMO for CS is being considered.

The current ongoing trials will hopefully provide sufficient information to definitively establish any potential indication for ECMO in CS (Table 3).

The EURO-SHOCK [94] was a large randomised trial conducted in Europe comparing ECMO vs. optimal medical therapy in patients with CS due to STEMI. The study aimed at enrolling 428 patients with CS following an acute coronary syndrome. Unfortunately, it was prematurely stopped after the inclusion of 32 patients due to slow recruitment [95].

The ANCHOR trial is an ongoing French multicentre study [96], including a total of 400 STEMI patients complicated by CS who will be randomly assigned to a combination of ECMO and IABP or optimal medical therapy.

The ECLS-SHOCK trial [97] is an international multicentre trial that will randomise 420 STEMI patients with severe-infarct-related cardiogenic shock undergoing early revascularisation with percutaneous coronary intervention or, alternatively, coronary artery bypass grafting, and optimal medical treatment will be randomly assigned to ECMO or optimal medical therapy.

## 6. Conclusions

Despite advances in technology and mechanical devices, CS is still associated with an extremely high rate of mortality, especially when caused by AMI. Given the lack of RCTs with an adequate sample size, there is a paucity of information on the correct management to adopt in CS complicating AMI. To date, the most important and effective measure in this subgroup of patients remains early revascularisation. Future efforts must be directed to collect more evidence in order to better identify the correct timing of initiation and select CS patients who may further benefit from inotropic therapies and advanced mechanical supports.

## Figures and Tables

**Table 1:** Definition of cardiogenic shock in clinical trials and guidelines.

Shock	Iabp Shock II	Culprit-Shock	Esc Heart Failure Guidelines	Orbi Risk Score
■ SBP <90 mmHg for ≥30 min or Support to maintain SBP ≥ 90 mmHg and ■ II. Endorgan hypoperfusion (urine output < 30 mL/h or cool extremities and heart rate > 60 b.p.m.) ■ Haemodynamic criteria *: CI of ≤ 2.2 L/min/m^2^ and PCWP ≥ 15 mmHg	■ SBP < 90 mmHg for ≥30 min or catecholamines to maintain SBP > 90 mmHg and ■ Clinical pulmonary congestion and ■ Impaired endorgan perfusion with at least one of the following criteria: Altered mental status Cold/clammy skin and extremities Urine output < 30 mL/h Lactate > 2.0 mmol/L	■ Planned early revascularization by PCI ■ Multivessel coronary artery disease defined as >70% stenosis in at least two major vessels (≥2 mm diameter) with identifiable culprit lesion SBP < 90 mmHg for >30 min or Catecholamines required to maintain SBP > 90 mmHg ■ Pulmonary congestion ■ Impaired organ perfusion with at least one of the following criteria: Altered mental status Cold/clammy skin and extremities Urine output < 30 mL/h Lactate > 2.0 mmol/L	SBP < 90 mmHg with adequate volume and clinical or laboratory signs of hypoperfusion *Clinical hypoperfusion*: Cold extremities, oliguria, mental confusion, dizziness, and narrow pulse pressure. *Laboratory hypoperfusion*: Metabolic acidosis Elevated lactate Elevated creatinine	SBP ≤ 90 mmHg for >30 min following exclusion of hypovolaemia, with clinical evidence of hypoperfusion, inotrope dependence, or mechanical left ventricular support to correct this situation

* Not required in anterior infarction or if pulmonary congestion in chest X-ray: CI, cardiac index; ESC, European Society of Cardiology; PCI, percutaneous coronary intervention; PCWP, pulmonary capillary wedge pressure; SBP, systolic blood pressure.

**Table 2:** Technical features of current percutaneous mechanical support devices for cardiogenic shock.

	Iabp	Impella	Tandemheart	Ecmo
Structure	Pneumatic pump	Axial pump	Centrifugal pump	Centrifugal pump
Cannula size	7–8 Fr	14 Fr (Impella CP) 21 Fr (Impella 5.0)	21 Fr venous (inflow) 12–19 Fr arterial (outflow)	17–21 Fr venous (inflow) 14–19 Fr artery (outflow)
Insertion/Placement	Femoral artery	Femoral artery (CP) Transaortic/Transubclavian (for Impella 5.5)	Femoral artery Femoral vein for left atrial access	Femoral artery Femoral vein
Cardiac output	0.5–0.8 L/min	3.7–5 L/min	4.5 L/min	>4.5 L/min
Max. no. of implantation days	weeks	5–7 day (up to 30 days for Impella 5.5)	14 day	weeks
Complexity of insertion	low	Medium (High for Impella 5.0)	high	Medium
Risk of hemolysis	low	Moderate	Moderate	Moderate
Risk of complications: (1) Ischemic (2) Hemorrhagic (3) Infectious	Low Low Low	Moderate (High for Impella 5.0) Moderate Low (moderate for Impella 5.0)	High High Moderate	High High Moderate

**Table 3:** Ongoing trials testing MCS in the setting of CS.

MCS	Trial	Study Population	Active Treatment and Comparator	Primary Study Endpoint
Impella CP^®^	DanGer Shock (77) NCT01633502	330 STEMI patients complicated by CS patients undergoing PCI	Impella CP vs. conventional circulatory support	death from all causes through 180 days
Impella CP^®^	STEMI-DTU (81) NCT03947619	668 patients with anterior STEMI	Impella CP placement prior to reperfusion with primary PCI vs. primary PCI alone	Infarct size (evaluated by cardiac magnetic resonance at 3–5 days after PCI)
VA ECMO	ANCHOR (97) NCT04184635	400 patients with STEMI complicated by CS	ECMO and IABP vs. optimal medical therapy	Treatment failure at Day 30
VA ECMO	ECLS-SHOCK (98) NCT03637205	420 patients with STEMI complicated by CS	ECMO vs. optimal medical therapy (in addition to early revascularization)	30-day mortality

## Data Availability

Not applicable.
